# Cross-Correlation Analysis of Monthly Google Search Volume and Suicide in Taiwan, 2012–2022

**DOI:** 10.1155/da/5515746

**Published:** 2025-06-05

**Authors:** Cheng-Fang Yen, Yu-Hsuan Lin, Ray C. Hsiao, Ying-Yeh Chen, Yi-Lung Chen

**Affiliations:** ^1^Department of Psychiatry, Kaohsiung Medical University Hospital, Kaohsiung Medical University, Kaohsiung, Taiwan; ^2^Department of Psychiatry, School of Medicine, College of Medicine, Kaohsiung Medical University, Kaohsiung, Taiwan; ^3^College of Professional Studies, National Pingtung University of Science and Technology, Pingtung, Taiwan; ^4^Institute of Population Health Sciences, National Health Researches Institutes, Zhunan, Miaoli County, Taiwan; ^5^Department of Psychiatry, National Taiwan University Hospital, Taipei, Taiwan; ^6^Department of Psychiatry, College of Medicine, National Taiwan University, Taipei, Taiwan; ^7^Institute of Health Behaviors and Community Sciences, College of Public Health, National Taiwan University, Taipei, Taiwan; ^8^Department of Psychiatry and Behavioral Sciences, University of Washington School of Medicine, Seattle, Washington, USA; ^9^Department of Psychiatry, Seattle Children's, Seattle, Washington, USA; ^10^Taipei City Psychiatric Center, Taipei City Hospital, Taipei City, Taiwan; ^11^Institute of Public Health, School of Medicine, National Yang Ming Chiao Tung University, Taipei City, Taiwan; ^12^Department of Healthcare Administration, Asia University, Taichung, Taiwan

**Keywords:** Google Trends, suicide, time-series analysis

## Abstract

**Background:** The present study investigated the 1-month, 2-month, and 3-month prospective associations of Google search terms with suicide in Taiwan from 2012 to 2022.

**Methods:** We analyzed daily suicide data from Taiwan's Cause of Death Statistics between January 1, 2012, and December 31, 2022. Data on Google search volumes for 37 terms related to suicide-related, socioeconomic status, familial problems, and physical and psychiatric problems were extracted from Google Trends. Cross-correlation coefficients between monthly Google search term volumes and monthly suicide were calculated at lags of 3 months (lag-3), 2 months (lag-2), and 1 month (lag-1).

**Results:** The monthly Google search volumes of two terms, “pain” and “Taiwan economy”, positively predicted monthly suicide in the total population. The search term “hypnotic” lag-3 negatively correlated with monthly suicide in the population aged ≥65. The search term “allergy” lag-1 positively correlated with monthly suicide in the population aged ≥65.

**Conclusions:** The monthly Google search terms of “pain” and “Taiwan economy” positively correlated with monthly suicide. The search terms “hypnotic” and “allergy” negatively and positively correlated with monthly suicide in the population aged ≥65, respectively. These terms may enable more accurate forecasting of future suicides.

## 1. Introduction

More than 7,00,000 people die due to suicide every year [1]. Suicide is the second leading cause of death among Taiwanese people aged 15–44 years in 2022, only second to traffic accidents and cancer among people aged 15–24 and 25–44, respectively [2]. Suicide not only causes the premature loss of human life but also affects the mental health of family members, friends, and peers exposed to it [3, 4]. Suicide also has social and economic burdens [5]. Factors that can predict suicide must be investigated to improve the effectiveness of suicide prevention plans and policies. A meta-analysis of 365 studies investigating the risk factors for suicidal thoughts and behaviors demonstrated that prior suicide ideation, hopelessness, depression, abuse history, and anxiety were the five strongest predictors of suicidal thoughts and behaviors. However, the predictive power of these factors is low, indicating that a broad range of factors may be associated with suicide [6].

With the popularity of the Internet, Internet search has become a convenient way for people to get answers to their questions and project their thoughts. The volume of Internet searches is being used as a social indicator; Internet search query data are not only used to analyze consumer behaviors [7] but also to predict the outbreak of respiratory infections [8], and unemployment [9]. Studies have confirmed the prediction of Internet searches for suicide and suicide methods (such as hanging, pesticide, poison, and jumping from the high) [10–18] and mental health problems (such as depression, insomnia, anxiety disorder, alcohol use, and bipolar disorder) [10, 14, 18] for suicide. Studies have also demonstrated significant associations of Internet searches for physical problems (such as allergy and pain) [14, 18], relationship problems (such as relationship breakup, domestic violence, and divorce) [14, 18], and socioeconomic difficulties (such as no money, being laid off, and lawsuits) [15, 17, 18] with suicide.

As one of the pioneer studies, Yang et al. [18] examined the temporal relationships of Google search volumes of 37 terms and suicide rates in Taipei City, Taiwan, during the period from 2004 to 2009 and demonstrated that a set of Google search terms, including “major depression,” “bipolar disorder,” “anxiety disorder,” “insomnia,” “divorce,” “domestic violence,” “Taiwan economy,” “religious belief,” and “complete guide of suicide” are positively associated with the rates of all suicide or subtype of suicide in the total or sex-specific populations. The findings of Yang et al. [18] provide important knowledge for the socio–cultural context of suicide and the references for the development of suicide prevention strategies in Taiwan. However, the Internet access rate of the Taiwanese population increased from 71% in 2009 to 84.7% in 2023 [19]. The rates of suicide have also significantly changed in the last decade compared with those before 2010 [20]. The temporal relationships between Google search term volumes and suicide should be re-examined using the last decade of data from Taiwan. Moreover, Yang et al. [18] examined the temporal relationships of Google search terms and suicide rates using the data from a single city in Taiwan. The use of Taiwan-wide data for analysis will facilitate the development of sex- and age-specific suicide prevention strategies.

The present study investigated the 1-month, 2-month, and 3-month prospective associations of Google search volumes of 37 terms for suicide-related, socioeconomic status, familial problems, and physical and psychiatric problems with suicide in Taiwan from 2012 to 2022. These 37 Google search terms were first examined in the study by Yang et al. [18]. We hypothesized that the Google search volumes of 37 terms have predictive effects on suicide one to 3 months later. Through this research, we provided evidence-based recommendations for the Taiwanese government and mental health professionals regarding early detection and management strategies for suicide among Taiwanese people.

## 2. Methods

### 2.1. Suicide Death

Suicide mortality data were obtained from Taiwan's national cause-of-death mortality database. International Classification of Diseases, 10th Revision codes X60–X84 were used to identify suicide deaths [21]. Monthly suicide counts for the period between 2012 and 2022 were retrieved. This study was approved by the Institutional Review Board of Taipei City Hospital Research Ethics Committee (TCHIRB-11012016).

### 2.2. Google Search Term Volumes

We leveraged data from Google Trends to gain insights into various societal aspects in Taiwan. We focused on monthly searches conducted in Traditional Chinese for 37 terms within the period from January 1, 2012 to August 26, 2023: 14 psychiatric terms (“suicide,” “major depression,” “bipolar disorder,” “schizophrenia,” “anxiety disorder,” “stress,” “illicit drugs,” “alcohol,” “drunkenness,” “alcohol abstinence,” “insomnia,” “hypnotics,” “antidepressant,” and “psychiatric service”); six medical terms (“asthma,” “allergy,” “pain,” “headache,” “cancer,” and “chronic illness”); five familial terms (“marriage,” “divorce,” “abuse,” “domestic violence,” and “relationship breakup”); eight socioeconomic terms (“job,” “unemployment,” “social welfare,” “social benefits,” “religious belief,” “stock market,” “Taiwan economy,” and “lawsuit”); and four suicide-related terms (“hanging,” “jumping from a height,” “charcoal burning,” and “complete guide of suicide”). In this study, no quotation marks (“ ”) or functions (+ or −) were used for Google Trends queries. Our approach follows the same rationale, as Google search volume is based on a normalized relative search volume (RSV), making single-term queries preferable for consistency. Table S1 presents the English Google search terms alongside their corresponding Chinese translations.

Google Trends is a valuable resource for understanding search trends because of the widespread popularity of Google Search in Taiwan, which had a market share exceeding 90% during our research period. Google Trends does not provide absolute search numbers; instead, it offers a relative search value that quantifies search activity for a specific term within a designated period and geographical region. This relative value is calculated by dividing each data point by the total search activity within the corresponding geographic and temporal scope, effectively allowing for a comparison of the relative popularity of terms. The resulting values are scaled on a range from 0 to 100, where a value of 100 represents the peak popularity of the term, and a value of 50 indicates that the term was half as popular during a specific time period in comparison to the given baseline.

### 2.3. Statistical Analysis

All analyses were conducted using R Statistical Software (version 4.3.2; R Foundation for Statistical Computing, Vienna, Austria). To explore the temporal relationship between suicide and Google search term volumes, we initially employed a seasonal autoregressive integrated moving average model (SARIMA) on monthly suicide and Google search term volumes data [15]. This approach was aimed at obtaining prewhitened time series by eliminating seasonality and autocorrelation. The SARIMA model, facilitated by the “bayesforecast” package [22], automatically fitted seasonal ARIMA models using Bayesian analysis, selecting those with the best fit based on the Bayesian information criterion (BIC). Subsequently, we applied the cross-correlation function (CCF) to assess their associations between Google search term volumes and 3 months preceding the suicide death (at lags 3–1), consistent with the time frame used by Yang et al. [18], as their study observed longitudinal associations within this period. Since 37 searching terms were examined, a *p*-value < 0.00135 (0.05/37) was considered to indicate statistical significance. To determine if the associations between suicide and Google search term volumes varied by sex and age groups, we conducted similar analyses with stratification.

## 3. Results

Between 2012 and 2022, Taiwan recorded a total of 40,941 suicide deaths, averaging 310 per month, with monthly figures ranging between 241 and 386. The number of suicide deaths was comparatively lower in the two periods (from 2013–2015 to 2020–2021).

### 3.1. Monthly Google Search Volume and Monthly Suicide in the Total Population

**Figure 1:**
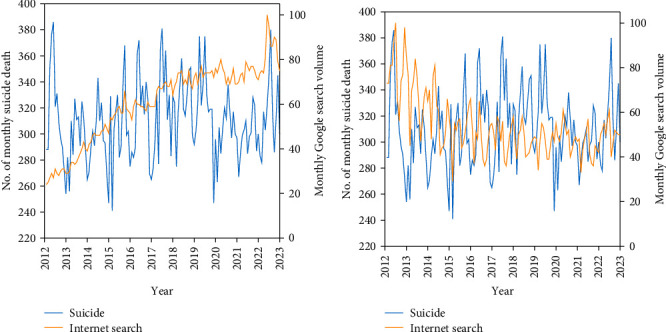
Trends of prewhitened monthly Google search volume of “pain” and “Taiwan economy” and monthly suicide rates among the total population in Taiwan between 2012 and 2022. (A) Monthly suicide deaths and Google search volume for the keyword “pain.” (B) Monthly suicide deaths and Google search volume for the keyword “Taiwan economy.” The left *y*-axis indicates the number of monthly suicide deaths, while the right *y*-axis indicates the monthly Google search volume.

All cross-correlation coefficients between the prewhitened monthly Google search volume terms and the filtered monthly suicide time series in the total population at lags 3, 2, and 1 are presented in Table 1 and Figure 1. Among the 37 search terms analyzed, the monthly Google search volume of two terms (“pain” and “Taiwan economy”) at lag-2 positively correlated with monthly suicide in the total population. These Google search terms belong to medical and socioeconomic terms, but not psychiatric, familial, or suicide-related terms. The search volume of the term “pain” had the strongest predictive effect, followed by “Taiwan economy.”

### 3.2. Monthly Google Search Volume and Monthly Suicide in the Male and Female Populations

All cross-correlation coefficients in males and females are presented in Table 2. Only “pain” significantly correlated with monthly suicide in males but not in females. Other search terms did not significantly correlate with monthly suicide in males or females.

### 3.3. Monthly Google Search Volume and Monthly Suicide in Various Age Groups

All cross-correlation coefficients in the groups of populations aged 18–44, aged 45–64, and aged ≥65 are presented in Table 3. The search term “hypnotic” lag-3 negatively correlated with monthly suicide in the population aged ≥65. The search term “allergy” lag-1 positively correlated with monthly suicide in the population aged ≥65. No search terms significantly correlate with monthly suicide in the populations aged 18–44 and aged 45–64.

## 4. Discussion

The present study found that the monthly Google search volumes of “pain” and “Taiwan economy” were prospectively correlated with an increase in monthly suicide in the total population. Further analysis found that the monthly Google search volume of “pain” positively correlates with suicide in males but not in females. The search terms “hypnotic” and “allergy” negatively and positively correlated with monthly suicide in the population aged ≥65, respectively.

The present study examined the cross-sectional and prospective associations of Google search volumes of 37 terms with suicide in Taiwan from 2012 to 2022. The study by Yang et al. [18] examined the associations of Google search volumes of the same 37 terms with suicide in Taipei City, Taiwan, during the period from 2004 to 2009 using multiple linear regression analysis. In line with the results of Yang et al. [18], the present study showed that the Google search volume of “Taiwan economy” was predictive of total suicide. Searching for “Taiwan economy” could suggest economic concerns and appear to forecast future suicide incidence. The result suggests that economic concerns have a significant impact on individuals' mental health. Contrary to the results of Yang et al. [18], the present study showed that the Google search volume of “pain” was predictive of total suicide. As presented in Figure 1, the Google search volume of “pain” increased persistently in the last decade, indicating an increasing number of Taiwanese individuals who suffer from pain. A review found that chronic pain increases sleep problems, poorer perceived mental health, hopelessness, perceived burdensomeness and thwarted belongingness, and then contributes to suicidality outcomes [23]. The results of this study show that pain is not only an important physical health issue for modern people, but it is also closely related to mental health.

Contrary to the results of the study by Yang et al. [18], the present study found that the search terms except for “pain” and “Taiwan economy” did not significantly correlate with suicide in the total population. Several reasons may account for the discrepancies between the results of the study by Yang et al. [18] and the present study. First, a strict statistical significance (*p* < 0.00135) was adopted to reduce the risk of false-positive outcomes arising from multiple comparisons. Second, the study by Yang et al. [18] examined suicide in the population of a single city (Taipei) in Taiwan, whereas the present study examined the suicide of the whole Taiwanese population. Third, the study by Yang et al. [18]examined Google search volumes and suicide during the period from 2004 to 2009, whereas the present study examined Google search volumes and suicide during the period from 2012 to 2022. Both the ubiquity of Internet search and suicide have changed in the last decade. Fourth, the variation in statistical methods across studies may partly explain the differing outcomes observed. Although both Yang et al. [18] and our study utilized CCF analysis, our approach included adjusting for potential seasonality and autocorrelation in Google search terms and suicide. Specifically, we employed seasonal ARIMA models to obtain prewhitened time series before conducting the CCF analysis, a step not undertaken by Yang et al. [18]. As a result, our findings may be less subject to confounding effects from seasonality and autocorrelation.

The medical terms “pain” and “allergy” positively predict suicide in the total population and the population aged ≥65, respectively. Although the elderly are less likely to search the Internet because they are unfamiliar with modern technology, their children, who are concerned about the health of the elderly, may search the Internet to gain knowledge about caring for the elderly. In Taiwanese culture, taking care of the elderly is one of the responsibilities of children to fulfill their filial piety [24]. Therefore, children may search for keywords such as pain, sleeping pills, and allergy for the sake of the elderly's health problems. Therefore, these keywords may represent the sleep and physical health problems that disturb the elderly. Pain and allergy can increase the risk of suicide via multiple biological and psychological pathways [23, 25]. Alternatively, this study found that “hypnotic” negatively correlated with monthly suicide in the population aged ≥65. Insomnia has been identified to increase the risk of suicide [26]. It is possible that searching “hypnotic” indicates the awareness of the negative consequences of insomnia and the motivation to manage insomnia. Thus, the risk of suicide decreases. However, further study is warranted to examine the association between the search term “hypnotic” and suicide.

Several psychiatric terms (such as “major depression” and “bipolar disorder”), familial terms (such as “divorce” and “domestic violence”), and suicide-related terms (such as “complete guide of suicide”) that predicted suicide in the study by Yang et al. [18] did not significantly predict suicide in the present study. However, the nonsignificance of these previously-reported correlations does not mean that these disorders are not predictive of suicide risk anymore. Further study is needed to examine the roles of new pharmacological interventions developed for treating major depression and bipolar disorder, de-stigmatization campaigns toward mental illnesses in the early 21st century, and the policy of prevention and early detection of domestic violence for the correlations of Google search for psychiatric and familial terms with suicide [27–29].

### 4.1. Limitations

This study examined the prediction of monthly Google search term volumes for suicide in Taiwan with a new database and statistical approach. However, the present study has several limitations. First, the results of this study have limited generalizability because all Google searches analyzed in the study were conducted in Traditional Chinese within Taiwan. Second, this study might not adequately adjust for potential sociodemographic confounders because Google Trends does not provide detailed information on user demographics. As a result, we could not incorporate sex- or age-specific search-volume data into our analysis. Nevertheless, our study adopted an ecological study design, where the observational unit is a group rather than separate individuals for one or more study variables. It has been noted in the literature that certain confounders relevant at the individual level might not apply similarly in ecological studies. Third, the study could not determine what proportion of searches were generated by individuals at risk of suicide, their friends and families, medical providers, or internet users influenced by media portrayals about suicide. Fourth, the present study examined the prediction of 37 Google search terms proposed in the study by Yang et al. [18] for suicide in Taiwan. Further study is needed to include more Google search terms and examine their predictive effects on suicide. Fifth, those who searched Google are not necessarily those who later died by suicide. This study explored the Google search volumes as a correlate of particular trends of popular information at a societal level. Finally, it has been proposed that suicide is underreported in Taiwan. However, this situation may represent nondifferential misclassification because underreported suicide cases would be misclassified as nonsuicide cases, and such misclassification would not be influenced by Google searches. It has been reported that such a nondifferential misclassification outcome introduces additional random error, leading the study results toward the null [30].

### 4.2. Implications of the Study

The present study identified the monthly Google search volumes of “pain” and “Taiwan economy” that can positively predict monthly suicide in the total population of Taiwan. The present study also found that the search terms “hypnotic” and “allergy” significantly correlated with monthly suicide in the population aged ≥65. Through adequate modeling processes, these terms may enable a high-precision forecast of future suicide before official data on suicide deaths are published with a substantial time lag. Moreover, the suicide-predictive search terms can serve as the basis for policymakers to develop effective online interventions for suicide prevention.

## Figures and Tables

**Table 1:** Results with not statistically significant of cross-correlation analysis of prewhitened monthly Google search volume and monthly suicide rate in the total population of Taiwan between 2012 and 2022.

Variable	Lag-3	Lag-2	Lag-1
Psychiatric terms			
Suicide	−0.080 (0.356)	0.064 (0.459)	0.120 (0.169)
Major depression	−0.030 (0.732)	−0.056 (0.520)	0.010 (0.911)
Bipolar disorder	−0.086 (0.321)	0.069 (0.428)	−0.019 (0.826)
Schizophrenia	−0.027 (0.754)	−0.100 (0.249)	−0.042 (0.629)
Anxiety disorder	−0.051 (0.555)	0.080 (0.359)	0.133 (0.126)
Stress	−0.049 (0.575)	0.084 (0.332)	−0.139 (0.110)
Illicit drugs	−0.014 (0.872)	−0.003 (0.972)	0.000 (0.998)
Alcohol	−0.069 (0.431)	−0.112 (0.197)	−0.169 (0.053)
Drunkenness	−0.142 (0.104)	−0.021 (0.814)	−0.084 (0.335)
Alcohol abstinence	0.077 (0.376)	0.179 (0.040)	0.095 (0.276)
Insomnia	−0.024 (0.782)	0.207 (0.018)	0.094 (0.278)
Hypnotics	−0.160 (0.065)	0.082 (0.345)	0.038 (0.665)
Antidepressant	0.011 (0.898)	0.123 (0.157)	−0.097 (0.265)
Psychiatric service	0.006 (0.949)	0.029 (0.738)	0.009 (0.915)
Medical terms			
Asthma	−0.068 (0.435)	−0.143 (0.101)	−0.135 (0.120)
Allergy	0.099 (0.253)	0.047 (0.588)	0.234 (0.007)
Pain	0.013 (0.881)	0.322 (<0.001)*⁣*^*∗*^	−0.012 (0.894)
Headache	0.053 (0.545)	0.098 (0.258)	0.025 (0.772)
Cancer	0.160 (0.066)	−0.069 (0.425)	−0.011 (0.898)
Chronic illness	0.078 (0.370)	0.019 (0.825)	−0.059 (0.495)
Familial terms			
Marriage	0.050 (0.567)	−0.106 (0.224)	−0.040 (0.644)
Divorce	0.031 (0.726)	0.014 (0.868)	−0.031 (0.725)
Abuse	0.048 (0.578)	0.041 (0.635)	0.075 (0.391)
Domestic violence	−0.061 (0.483)	0.101 (0.247)	−0.054 (0.537)
Relationship breakup	−0.031 (0.718)	−0.091 (0.298)	−0.022 (0.800)
Socioeconomic terms			
Job	0.061 (0.483)	0.150 (0.085)	0.139 (0.109)
Unemployment	0.019 (0.824)	0.011 (0.901)	−0.035 (0.689)
Social welfare	−0.004 (0.961)	−0.074 (0.398)	−0.061 (0.484)
Social benefits	0.025 (0.773)	0.166 (0.056)	−0.069 (0.426)
Religious belief	0.031 (0.723)	0.021 (0.808)	−0.110 (0.207)
Stock market	0.023 (0.788)	0.010 (0.912)	−0.035 (0.685)
Taiwan economy	−0.061 (0.485)	0.284 (0.001)*⁣*^*∗*^	−0.079 (0.366)
Lawsuit	−0.053 (0.543)	0.150 (0.086)	−0.019 (0.829)
Pro-suicide terms			
Hanging	0.068 (0.434)	−0.110 (0.206)	−0.030 (0.728)
Jumping from a high	−0.044 (0.611)	−0.034 (0.697)	−0.046 (0.594)
Charcoal burning	−0.152 (0.081)	−0.150 (0.084)	0.073 (0.401)
Complete guide of suicide	0.013 (0.882)	−0.003 (0.973)	−0.026 (0.768)

*Note:* The data are shown as cross-correlation coefficient and *p*-value.

*⁣* ^*∗*^ *p*  < 0.00135.

**Table 2:** Cross-correlation analysis of prewhitened monthly Google search volume and monthly suicide rate in Taiwan between 2012 and 2022, stratified by sex.

Variable	Male			Female		
	Lag-3	Lag-2	Lag-1	Lag-3	Lag-2	Lag-1
Psychiatric terms						
Suicide	−0.064 (0.461)	0.029 (0.736)	0.128 (0.142)	−0.066 (0.446)	0.090 (0.302)	0.051 (0.561)
Major depression	0.000 (0.996)	−0.023 (0.795)	0.009 (0.915)	−0.062 (0.473)	−0.079 (0.364)	0.013 (0.884)
Bipolar disorder	−0.091 (0.297)	0.061 (0.480)	−0.038 (0.664)	−0.044 (0.617)	0.060 (0.492)	0.018 (0.839)
Schizophrenia	−0.071 (0.414)	−0.082 (0.345)	−0.022 (0.797)	0.050 (0.566)	−0.090 (0.300)	−0.060 (0.489)
Anxiety disorder	−0.072 (0.407)	0.061 (0.483)	0.068 (0.436)	0.010 (0.910)	0.085 (0.328)	0.184 (0.034)
Stress	−0.101 (0.245)	0.090 (0.302)	−0.069 (0.427)	0.052 (0.552)	0.040 (0.643)	−0.175 (0.044)
Illicit drugs	−0.029 (0.741)	−0.035 (0.690)	0.006 (0.945)	0.015 (0.861)	0.052 (0.546)	−0.008 (0.929)
Alcohol	0.018 (0.840)	−0.161 (0.064)	−0.166 (0.056)	−0.171 (0.049)	0.009 (0.920)	−0.105 (0.228)
Drunkenness	−0.062 (0.477)	0.001 (0.991)	−0.086 (0.323)	−0.205 (0.019)	−0.051 (0.558)	−0.049 (0.575)
Alcohol abstinence	0.099 (0.254)	0.178 (0.041)	0.080 (0.358)	0.011 (0.897)	0.111 (0.202)	0.088 (0.311)
Insomnia	−0.045 (0.603)	0.184 (0.034)	0.106 (0.225)	0.024 (0.787)	0.161 (0.064)	0.046 (0.598)
Hypnotics	−0.117 (0.178)	0.087 (0.317)	0.008 (0.927)	−0.149 (0.087)	0.043 (0.624)	0.082 (0.348)
Antidepressant	0.009 (0.915)	0.095 (0.273)	−0.069 (0.429)	0.021 (0.810)	0.115 (0.186)	−0.098 (0.259)
Psychiatric service	−0.003 (0.971)	0.068 (0.432)	−0.037 (0.670)	0.020 (0.818)	−0.041 (0.635)	0.095 (0.275)
Medical terms						
Asthma	−0.062 (0.473)	−0.075 (0.387)	−0.171 (0.049)	−0.058 (0.508)	−0.189 (0.030)	−0.027 (0.758)
Allergy	0.035 (0.688)	0.038 (0.663)	0.224 (0.010)	0.158 (0.070)	0.045 (0.605)	0.151 (0.084)
Pain	−0.033 (0.701)	0.293 (0.001)*⁣*^*∗*^	−0.023 (0.794)	0.108 (0.214)	0.193 (0.027)	0.020 (0.815)
Headache	−0.005 (0.954)	0.061 (0.482)	0.089 (0.307)	0.124 (0.155)	0.110 (0.208)	−0.090 (0.299)
Cancer	0.122 (0.159)	−0.078 (0.368)	−0.063 (0.467)	0.153 (0.079)	−0.022 (0.798)	0.080 (0.359)
Chronic illness	0.067 (0.443)	0.086 (0.321)	−0.069 (0.430)	0.062 (0.479)	−0.091 (0.294)	−0.019 (0.823)
Familial terms						
Marriage	0.043 (0.621)	−0.100 (0.252)	0.037 (0.674)	0.072 (0.407)	−0.104 (0.234)	−0.076 (0.383)
Divorce	0.021 (0.807)	0.055 (0.530)	−0.116 (0.183)	0.043 (0.625)	−0.063 (0.469)	0.131 (0.132)
Abuse	0.055 (0.525)	0.010 (0.910)	0.057 (0.511)	0.027 (0.756)	0.066 (0.451)	0.072 (0.410)
Domestic violence	−0.034 (0.699)	0.125 (0.151)	−0.100 (0.252)	−0.076 (0.383)	0.018 (0.835)	0.042 (0.633)
Relationship breakup	−0.010 (0.911)	−0.093 (0.285)	−0.050 (0.565)	−0.053 (0.543)	−0.051 (0.562)	0.032 (0.715)
Socioeconomic terms						
Job	0.051 (0.557)	0.152 (0.081)	0.078 (0.371)	0.056 (0.524)	0.097 (0.264)	0.178 (0.040)
Unemployment	0.031 (0.722)	0.052 (0.547)	−0.040 (0.648)	−0.013 (0.882)	−0.058 (0.504)	−0.015 (0.864)
Social welfare	−0.024 (0.782)	−0.069 (0.427)	−0.064 (0.462)	0.030 (0.727)	−0.046 (0.596)	−0.024 (0.783)
Social benefits	0.009 (0.919)	0.170 (0.051)	−0.082 (0.347)	0.046 (0.601)	0.094 (0.279)	−0.013 (0.878)
Religious belief	0.076 (0.386)	−0.036 (0.677)	−0.022 (0.797)	−0.049 (0.575)	0.086 (0.322)	−0.198 (0.023)
Stock market	0.011 (0.900)	0.059 (0.501)	−0.039 (0.650)	0.025 (0.772)	−0.070 (0.419)	−0.019 (0.827)
Taiwan economy	−0.107 (0.221)	0.222 (0.011)	−0.068 (0.432)	0.040 (0.646)	0.252 (0.004)	−0.062 (0.480)
Lawsuit	−0.088 (0.315)	0.152 (0.081)	−0.015 (0.864)	0.030 (0.732)	0.090 (0.301)	0.002 (0.981)
Pro-suicide terms						
Hanging	0.060 (0.493)	−0.043 (0.621)	−0.010 (0.907)	0.048 (0.581)	−0.168 (0.054)	−0.044 (0.615)
Jumping from a high	−0.039 (0.652)	−0.031 (0.722)	−0.047 (0.591)	−0.032 (0.714)	−0.025 (0.770)	−0.016 (0.855)
Charcoal burning	−0.118 (0.175)	−0.156 (0.072)	0.104 (0.232)	−0.141 (0.105)	−0.075 (0.389)	0.006 (0.946)
Complete guide of suicide	0.017 (0.842)	0.041 (0.639)	−0.062 (0.473)	−0.006 (0.944)	−0.068 (0.432)	0.034 (0.694)

*Note:* The data are shown as cross-correlation coefficient and *p*-value.

*⁣* ^*∗*^ *p*  < 0.00135.

**Table 3:** Cross-correlation analysis of prewhitened monthly Google search volume and monthly suicide rate in Taiwan between 2012 and 2022, stratified by age groups.

Variable	Age 18–44			Age 45–64			Age ≥ 65		
	Lag-3	Lag-2	Lag-1	Lag-3	Lag-2	Lag-1	Lag-3	Lag-2	Lag-1
Psychiatric terms									
Suicide	−0.180 (0.038)	0.067 (0.441)	0.073 (0.401)	0.009 (0.919)	−0.001 (0.990)	0.088 (0.310)	−0.086 (0.325)	0.030 (0.729)	0.037 (0.671)
Major depression	−0.083 (0.341)	0.041 (0.635)	0.123 (0.159)	0.112 (0.197)	0.043 (0.620)	0.005 (0.958)	−0.051 (0.560)	−0.230 (0.008)	−0.042 (0.629)
Bipolar disorder	−0.086 (0.326)	0.025 (0.771)	0.038 (0.661)	−0.069 (0.425)	0.165 (0.059)	−0.065 (0.453)	−0.031 (0.721)	−0.071 (0.413)	−0.007 (0.939)
Schizophrenia	0.028 (0.745)	−0.071 (0.412)	−0.018 (0.835)	−0.056 (0.519)	−0.063 (0.466)	−0.058 (0.508)	0.019 (0.824)	−0.023 (0.788)	−0.001 (0.991)
Anxiety disorder	−0.083 (0.339)	0.098 (0.260)	0.120 (0.169)	−0.048 (0.579)	0.009 (0.917)	−0.079 (0.367)	−0.046 (0.600)	−0.009 (0.921)	0.208 (0.017)
Stress	−0.125 (0.152)	0.155 (0.075)	0.073 (0.401)	0.080 (0.360)	0.093 (0.286)	−0.179 (0.039)	−0.010 (0.905)	−0.033 (0.701)	−0.123 (0.159)
Illicit drugs	0.057 (0.510)	−0.001 (0.991)	−0.013 (0.884)	−0.009 (0.920)	0.005 (0.951)	0.011 (0.896)	−0.063 (0.467)	−0.004 (0.959)	0.048 (0.582)
Alcohol	−0.018 (0.835)	−0.030 (0.726)	−0.034 (0.695)	−0.073 (0.399)	−0.001 (0.993)	−0.163 (0.061)	−0.020 (0.822)	−0.224 (0.010)	−0.163 (0.061)
Drunkenness	−0.102 (0.241)	0.087 (0.319)	−0.033 (0.706)	−0.024 (0.782)	0.036 (0.678)	−0.032 (0.710)	−0.121 (0.165)	−0.140 (0.107)	−0.076 (0.386)
Alcohol abstinence	−0.046 (0.597)	0.009 (0.920)	0.127 (0.144)	0.210 (0.016)	0.209 (0.016)	0.017 (0.849)	−0.031 (0.722)	0.106 (0.223)	0.037 (0.669)
Insomnia	−0.097 (0.263)	0.063 (0.471)	0.121 (0.164)	0.010 (0.907)	0.128 (0.140)	0.032 (0.717)	−0.043 (0.621)	0.216 (0.013)	0.043 (0.622)
Hypnotic	0.030 (0.729)	0.051 (0.560)	0.000 (0.999)	−0.071 (0.415)	−0.001 (0.991)	−0.006 (0.946)	−0.287 (<0.001)*⁣*^*∗*^	0.139 (0.111)	0.069 (0.427)
Antidepressant	0.031 (0.721)	0.058 (0.502)	−0.086 (0.322)	−0.040 (0.644)	0.178 (0.040)	−0.021 (0.806)	0.025 (0.774)	−0.002 (0.986)	−0.127 (0.144)
Psychiatric service	−0.039 (0.655)	0.085 (0.331)	−0.055 (0.527)	0.130 (0.135)	0.032 (0.715)	−0.043 (0.622)	−0.143 (0.100)	−0.145 (0.096)	0.055 (0.531)
Medical terms									
Asthma	0.036 (0.676)	−0.090 (0.300)	0.017 (0.843)	−0.024 (0.779)	−0.059 (0.501)	−0.142 (0.102)	−0.106 (0.224)	−0.156 (0.073)	−0.140 (0.107
Allergy	0.025 (0.771)	0.141 (0.106)	0.036 (0.683)	0.101 (0.246)	−0.021 (0.807)	0.076 (0.384)	0.058 (0.508)	−0.030 (0.726)	0.429 (<0.001)*⁣*^*∗*^
Pain	0.030 (0.728)	0.188 (0.030)	−0.044 (0.609)	−0.029 (0.743)	0.228 (0.009)	−0.064 (0.462)	0.011 (0.898)	0.138 (0.112)	0.136 (0.118)
Headache	−0.043 (0.619)	0.140 (0.108)	0.052 (0.550)	0.053 (0.543)	0.019 (0.828)	−0.002 (0.984)	0.111 (0.200)	0.066 (0.449)	0.058 (0.509)
Cancer	0.149 (0.087)	−0.045 (0.605)	0.003 (0.973)	0.151 (0.083)	−0.014 (0.869)	−0.052 (0.550)	−0.001 (0.992)	−0.098 (0.260)	0.011 (0.903)
Chronic diseases	0.059 (0.499)	0.010 (0.907)	−0.073 (0.402)	0.002 (0.985)	0.068 (0.436)	−0.035 (0.689)	0.122 (0.160)	−0.037 (0.671)	−0.007 (0.938)
Familial terms									
Marriage	0.114 (0.190)	−0.056 (0.523)	−0.007 (0.937)	0.014 (0.875)	−0.059 (0.494)	0.018 (0.833)	0.058 (0.507)	−0.125 (0.152)	−0.008 (0.931)
Divorce	−0.013 (0.879)	−0.031 (0.721)	0.014 (0.871)	−0.036 (0.683)	−0.039 (0.655)	−0.033 (0.701)	0.077 (0.374)	0.127 (0.144)	−0.016 (0.859)
Abuse	0.048 (0.580)	0.064 (0.466)	0.247 (0.005)	0.069 (0.427)	0.040 (0.645)	−0.059 (0.501)	−0.036 (0.677)	−0.023 (0.790)	0.025 (0.776)
Domestic violence	−0.025 (0.772)	0.121 (0.164)	0.031 (0.723)	−0.079 (0.362)	0.059 (0.494)	−0.071 (0.414)	−0.007 (0.933)	0.022 (0.803)	−0.035 (0.686)
Relationship breakup	−0.064 (0.459)	−0.089 (0.305)	0.109 (0.212)	0.008 (0.927)	0.049 (0.577)	−0.111 (0.201)	−0.013 (0.879)	−0.142 (0.102)	−0.023 (0.796)
Socioeconomic terms									
Job	0.086 (0.321)	0.078 (0.368)	0.135 (0.120)	0.011 (0.897)	0.083 (0.341)	0.058 (0.502)	0.029 (0.741)	0.174 (0.046)	0.132 (0.130)
Unemployment	0.082 (0.349)	0.014 (0.868)	−0.023 (0.793)	0.045 (0.603)	−0.019 (0.826)	−0.008 (0.923)	−0.075 (0.389)	0.056 (0.521)	−0.024 (0.781)
Social welfare	0.032 (0.711)	0.089 (0.304)	0.015 (0.860)	−0.019 (0.825)	−0.125 (0.150)	−0.174 (0.046)	−0.025 (0.775)	−0.121 (0.164)	0.038 (0.659)
Social benefits	0.081 (0.350)	0.136 (0.119)	0.025 (0.777)	−0.032 (0.715)	0.129 (0.139)	−0.117 (0.178)	−0.017 (0.843)	0.052 (0.548)	−0.038 (0.662)
Religious belief	−0.010 (0.910)	0.200 (0.022)	−0.093 (0.284)	0.055 (0.531)	−0.054 (0.536)	−0.134 (0.122)	0.041 (0.640)	−0.075 (0.386)	0.029 (0.736)
Stock market	0.048 (0.578)	−0.041 (0.637)	−0.050 (0.565)	−0.007 (0.936)	0.031 (0.725)	0.059 (0.495)	0.065 (0.453)	0.094 (0.278)	−0.050 (0.563)
Taiwan economy	−0.008 (0.931)	0.230 (0.008)	−0.063 (0.467)	−0.065 (0.453)	0.246 (0.005)	−0.082 (0.346)	−0.053 (0.544)	0.103 (0.239)	−0.021 (0.811)
Lawsuit	−0.076 (0.381)	0.226 (0.009)	0.044 (0.614)	−0.033 (0.703)	0.035 (0.684)	−0.125 (0.152)	−0.045 (0.602)	0.015 (0.862)	0.035 (0.686)
Pro-suicide terms									
Hanging	0.057 (0.515)	−0.104 (0.231)	0.026 (0.767)	0.125 (0.150)	−0.022 (0.798)	0.082 (0.349)	−0.036 (0.678)	−0.066 (0.447)	−0.146 (0.094)
Jumping from a high	−0.112 (0.200)	0.028 (0.745)	−0.070 (0.418)	0.068 (0.438)	0.002 (0.983)	0.007 (0.939)	−0.061 (0.482)	−0.111 (0.203)	−0.047 (0.592)
Charcoal burning	−0.164 (0.059)	0.030 (0.732)	0.109 (0.209)	−0.031 (0.724)	−0.061 (0.483)	0.067 (0.444)	−0.086 (0.323)	−0.253 (0.004)	0.016 (0.854)
Complete guide of suicide	−0.016 (0.853)	0.004 (0.963)	−0.051 (0.561)	0.004 (0.968)	−0.004 (0.961)	−0.020 (0.816)	0.058 (0.506)	0.013 (0.878)	0.059 (0.494)

*Note:* The data are shown as cross-correlation coefficient and *p*-value.

*⁣* ^*∗*^ *p*  < 0.00135.

## Data Availability

The data that supports the findings of this study are available from the Health and Welfare Data Science Center, Ministry of Health. Restrictions apply to the availability of these data, which were used under license for this study. The data are available from the authors with the permission of the Health and Welfare Data Science Center, Ministry of Health.

## Supporting Information

Supporting Information Table S1: English and corresponding Chinese Google search terms.

## References

[1] World Health Organization (2023). Suicide. https://www.who.int/news-room/fact-sheets/detail/suicide.

[2] Ministry of Health and Welfare Taiwan (2023). National Cause of Death Survey in 2022. https://www.mohw.gov.tw/cp-16-74869-1.html.

[3] Andriessen K., Rahman B., Draper B., Dudley M., Mitchell P. B. (2017). Prevalence of Exposure to Suicide: A Meta-Analysis of Population-Based Studies. *Journal of Psychiatric Research*.

[4] Lee H., Kim M. J., Hong M. (2022). Effect of Suicidal Loss on Bereaved Individuals’ Suicidal Ideation: Structural Equation Model Using Attitudes Towards Suicide Scale and Moderation Effect of Interest in News Media. *Journal of Affective Disorders*.

[5] Law C.-K., Yip P. S. F., Chen Y.-Y. (2011). The Economic and Potential Years of Life Lost From Suicide in Taiwan, 1997–2007. *Journal of Crisis Intervention and Suicide Prevention*.

[6] Franklin J. C., Ribeiro J. D., Fox K. R. (2017). Risk Factors for Suicidal Thoughts and Behaviors: A Meta-Analysis of 50 Years of Research. *Psychological Bulletin*.

[7] Goel S., Hofman J. M., Lahaie S., Pennock D. M., Watts D. J. (2010). Predicting Consumer Behavior With Web Search. *Proceedings of the National Academy of Sciences*.

[8] Dugas A. F., Jalalpour M., Gel Y. (2013). Influenza Forecasting With Google Flu Trends. *PLoS ONE*.

[9] Askitas N., Zimmermann K. F. (2009). Google Econometrics and Unemployment Forecasting. *Applied Economics Quarterly*.

[10] Adler N., Cattuto C., Kalimeri K. (2019). How Search Engine Data Enhance the Understanding of Determinants of Suicide in India and Inform Prevention: Observational Study. *Journal of Medical Internet Research*.

[11] Arora V. S., Stuckler D., McKee M. (2016). Tracking Search Engine Queries for Suicide in the United Kingdom, 2004–2013. *Public Health*.

[12] Biddle L., Derges J., Mars B. (2016). Suicide and the Internet: Changes in the Accessibility of Suicide-Related Information Between 2007 and 2014. *Journal of Affective Disorders*.

[13] Biddle L., Donovan J., Hawton K. (2008). Suicide and the Internet. *BMJ*.

[14] Jimenez A., Santed-Germán M.-A., Ramos V. (2020). Google Searches and Suicide Rates in Spain, 2004–2013: Correlation Study. *JMIR Public Health and Surveillance*.

[15] Lee J.-Y. (2020). Search Trends Preceding Increases in Suicide: A Cross-Correlation Study of Monthly Google Search Volume and Suicide Rate Using Transfer Function Models. *Journal of Affective Disorders*.

[16] Lopez-Agudo L. A. (2020). The Association Between Internet Searches and Suicide in Spain. *Psychiatry Research*.

[17] Taira K., Hosokawa R., Itatani T. (2021). Predicting the Number of Suicides in Japan Using Internet Search Queries: Vector Autoregression Time Series Model. *JMIR Public Health and Surveillance*.

[18] Yang A. C., Tsai S.-J., Huang N. E., Peng C.-K. (2011). Association of Internet Search Trends With Suicide Death in Taipei City, Taiwan, 2004–2009. *Journal of Affective Disorders*.

[19] Taiwan Network Information Center (2023). Overall Internet Usage. https://report.twnic.tw/2023/TrendAnalysis_internetUsage.html.

[20] Chen Y.-Y., Yang C.-T., Pinkney E., Yip P. S. F. (2021). The Age-Period-Cohort Trends of Suicide in Hong Kong and Taiwan, 1979–2018. *Journal of Affective Disorders*.

[21] Chen Y.-Y., Chen F., Wu K. C.-C., Lu T.-H., Chi Y.-C., Yip P. S. F. (2023). Dynamic Reciprocal Relationships Between Traditional Media Reports, Social Media Postings, and Youth Suicide in Taiwan Between 2012 and 2021. *SSM - Population Health*.

[22] Haqbin S. R. K., Khan A. A. (2023). A Bayesian Prediction for the Total Fertility Rate of Afghanistan Using the Auto-Regressive Integrated Moving Average (ARIMA) Model. *Reliability: Theory & Applications*.

[23] Racine M. (2018). Chronic Pain and Suicide Risk: A Comprehensive Review. *Progress in Neuro-Psychopharmacology and Biological Psychiatry*.

[24] Chen W.-W., Wu C.-W., Yeh K.-H. (2015). How Parenting and Filial Piety Influence Happiness, Parent–Child Relationships and Quality of Family Life in Taiwanese Adult Children. *Journal of Family Studies*.

[25] Kõlves K., Barker E., De Leo D. (2015). Allergies and Suicidal Behaviors: A Systematic Literature Review. *Allergy and Asthma Proceedings*.

[26] Wu T. T., Zou Y. L., Xu K. D. (2023). Insomnia and Multiple Health Outcomes: Umbrella Review of Meta-Analyses of Prospective Cohort Studies. *Public Health*.

[27] Tang I.-C., Wu H.-C. (2008). An Exploration of Stigmatization and Destigmatization Toward Persons With Psychiatric Disabilities. *Journal of Disability Research*.

[28] Cheng Y.-H. A., Wu F.-C. F., Adamczyk A. (2016). Changing Attitudes Toward Homosexuality in Taiwan, 1995–2012. *Chinese Sociological Review*.

[29] Chang H.-Y., Lin C.-Y., Liu S.-Y. (2018). Three-Tier Five-Level Preventive Strategy for Domestic Violence and Sexual Violence Prevention in Taiwan. *Journal of the Formosan Medical Association*.

[30] Yland J. J., Wesselink A. K., Lash T. L., Fox M. P. (2022). Misconceptions About the Direction of Bias From Nondifferential Misclassification. *American Journal of Epidemiology*.

