# Supplementary material for: Cross-Correlation Analysis of Monthly Google Search Volume and Suicide in Taiwan, 2012–2022
Source: Depress Anxiety. 2025 Jun 5;2025:5515746. doi: 10.1155/da/5515746 (PMC12162158; doi:10.1155/da/5515746)
Supplement: Supporting Information — Table S1: English and corresponding Chinese Google search terms. [file 5515746.f1.docx]

Supplementary material for Prediction of Monthly Google Search Term Volumes for Suicide Rates in Taiwan, 2012–2022

*Supplementary Materials*

*Supplementary Table 1 English and Corresponding Chinese Google Search Terms*

Supplementary Table 1 English and Corresponding Chinese Google Search Terms

| **English** | **Chinese** | **English** | **Chinese** | **English** | **Chinese** |
| --- | --- | --- | --- | --- | --- |
| Suicide | 自殺 | Asthma | 氣喘 | Relief | 救濟 |
| Depression | 憂鬱症 | Allergies | 過敏 | Faith | 信仰 |
| Bipolar Disorder | 躁鬱症 | Pain | 痛 | Stock Market | 股市 |
| Schizophrenia | 精神分裂症 | Headache | 頭痛 | Taiwan's Economy | 台灣經濟 |
| Anxiety | 焦慮 | Cancer | 癌症 | Lawsuit | 訴訟 |
| Stress | 壓力 | Chronic Diseases | 慢性病 | Hanging | 上吊 |
| Drugs | 毒品 | Marriage | 婚姻 | Jumping from High Places | 跳樓 |
| Alcohol | 酒 | Divorce | 離婚 | Charcoal Burning | 燒炭 |
| Drunkenness | 酒醉 | Abuse | 虐待 | Complete Suicide Manual | 完全自殺手冊 |
| Alcohol Withdrawal | 戒酒 | Domestic Violence | 家暴 |  |  |
| Insomnia | 失眠 | Breakup | 分手 |  |  |
| Sleeping Pills | 安眠藥 | Work | 工作 |  |  |
| Antidepressants | 抗憂鬱藥 | Unemployment | 失業 |  |  |
| Psychiatry | 精神科 | Social Welfare | 社會福利 |  |  |
